# Identification of *Stim1* as a Candidate Gene for Exaggerated Sympathetic Response to Stress in the Stroke-Prone Spontaneously Hypertensive Rat

**DOI:** 10.1371/journal.pone.0095091

**Published:** 2014-04-15

**Authors:** Mohammed Zubaerul Ferdaus, Bing Xiao, Hiroki Ohara, Kiyomitsu Nemoto, Yuji Harada, Kathrin Saar, Norbert Hübner, Minoru Isomura, Toru Nabika

**Affiliations:** 1 Department of Functional Pathology, Shimane University School of Medicine, Izumo, Japan; 2 Department of Molecular Toxicology, School of Pharmaceutical Sciences, University of Shizuoka, Shizuoka, Japan; 3 Department of Surgical Pathology, Shimane University Hospital, Izumo, Japan; 4 Department of Experimental Genetics of Cardiovascular Diseases, Max-Delbrück-Center for Molecular Medicine, Berlin, Germany; Osaka University Graduate School of Medicine, Japan

## Abstract

The stroke-prone spontaneously hypertensive rat (SHRSP) is known to have exaggerated sympathetic nerve activity to various types of stress, which might contribute to the pathogenesis of severe hypertension and stroke observed in this strain. Previously, by using a congenic strain (called SPwch1.72) constructed between SHRSP and the normotensive Wistar-Kyoto rat (WKY), we showed that a 1.8-Mbp fragment on chromosome 1 (Chr1) of SHRSP harbored the responsible gene(s) for the exaggerated sympathetic response to stress. To further narrow down the candidate region, in this study, another congenic strain (SPwch1.71) harboring a smaller fragment on Chr1 including two functional candidate genes, *Phox2a* and *Ship2,* was generated. Sympathetic response to cold and restraint stress was compared among SHRSP, SPwch1.71, SPwch1.72 and WKY by three different methods (urinary norepinephrine excretion, blood pressure measurement by the telemetry system and the power spectral analysis on heart rate variability). The results indicated that the response in SPwch1.71 did not significantly differ from that in SHRSP, excluding *Phox2a* and *Ship2* from the candidate genes. As the stress response in SPwch1.72 was significantly less than that in SHRSP, it was concluded that the 1.2-Mbp congenic region covered by SPwch1.72 (and not by SPwch1.71) was responsible for the sympathetic stress response. The sequence analysis of 12 potential candidate genes in this region in WKY/Izm and SHRSP/Izm identified a nonsense mutation in the stromal interaction molecule 1 (*Stim1*) gene of SHRSP/Izm which was shared among 4 substrains of SHRSP. A western blot analysis confirmed a truncated form of STIM1 in SHRSP/Izm. In addition, the analysis revealed that the protein level of STIM1 in the brainstem of SHRSP/Izm was significantly lower when compared with WKY/Izm. Our results suggested that *Stim1* is a strong candidate gene responsible for the exaggerated sympathetic response to stress in SHRSP.

## Introduction

The sympathetic nervous system (SNS) has been implied to play a key role in the pathogenesis of hypertension [1]. In the classical physiology, SNS is thought particularly important in modulating cardiovascular functions to cope with the various stresses and environmental changes [2]. The stroke-prone spontaneously hypertensive rats (SHRSP) are known to be vulnerable to various types of stress, which might contribute to the pathogenesis of severe hypertension and stroke observed in this strain [3]–[5], and thus the genetic factors underlying this may provide us important clues to understand some aspects of the pathogenesis of hypertension in humans. To identify the genomic region controlling the exaggerated sympathetic response to stress in SHRSP, we constructed reciprocal congenic strains between SHRSP and the normotensive Wistar-Kyoto rat (WKY) and compared physiological stress responses among congenic strains. In the previous study, we found that SPwch1.72, a congenic strain carrying a 1.8-Mbp of WKY fragment of chromosome 1 (Chr1) on the SHRSP background, had significantly lower sympathetic response to stress than did SHRSP [5]. This finding indicated that a gene (or genes) responsible for exaggerated sympathetic response to stress in SHRSP was located in the 1.8-Mbp region on Chr1. In this region, several interesting candidate genes were found such as the paired mesoderm homeobox protein 2a (*Phox2a*) and the SH2 domain-containing inositol 5′-phosphatase (*Ship2*).

PHOX2A is a transcription factor regulating the expression of the tyrosine hydroxylase (Th) in noradrenergic neurons [6], [7]. PHOX2A may thus play a key role in the pathogenesis of hypertension through controlling the activity of SNS. SHIP2 is a phosphatase encoded by Inppl1, and was described to negatively control the activity of the phosphatidylinositol 3-kinase (PI3K) [8]. Marion et al. identified a missense variation, R1142C, in the Ship2 gene of the Goto-Kakizaki (GK) diabetic rat and SHR, which reduced the signals downstream of PI3K probably due to the increased SHIP2 activity [9]. We confirmed that SHRSP shared the same variation with GK rats and SHR (data not shown), and as the pathway including PI3K was involved in the signal transduction activated by several vasoactive substances [10], [11], functional changes in SHIP2 may be causally related to hypertension in SHR and SHRSP.

Based on such observation, we attempted to construct another congenic strain harboring a smaller fragment including these candidate genes. In this report, physiological evaluation of the stress responses in a new congenic strain (SPwch1.71) indicated that the region harboring Phox2a and Ship2 was not likely to have causative roles in the exaggerated stress response in SHRSP. In spite of that, further attempt to explore causative genes in the remaining candidate region succeeded to identify another strong candidate gene, the stromal interaction molecule 1 (Stim1).

## Materials and Methods

### Ethics Statement

All experimental protocols of animal studies were approved by the ethics committee for animal research of Shimane University (#IZ23-60 and #IZ23-63).

### Animal Studies

**Figure 1:**
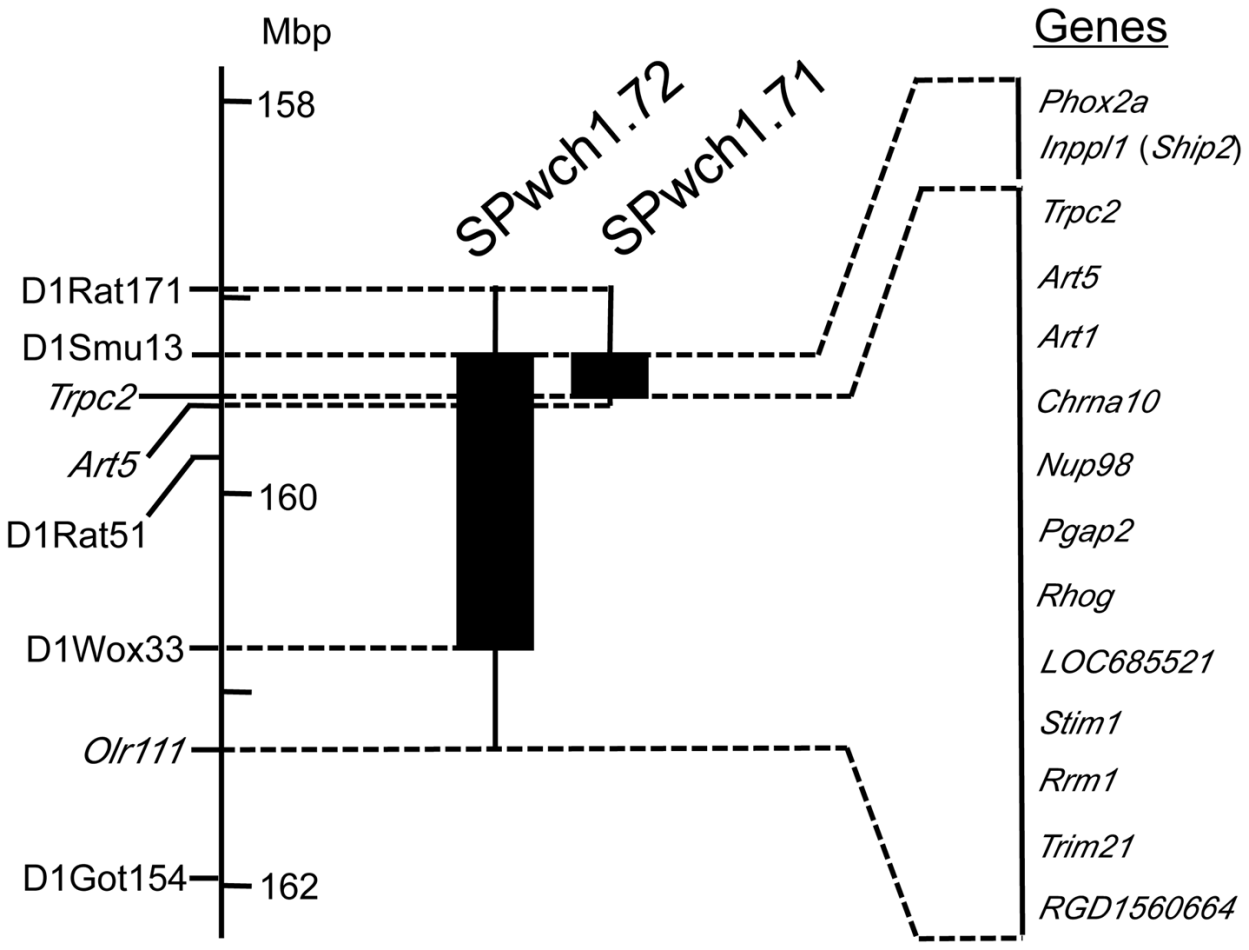
Genetic map of the congenic region and candidate genes located in the region. Closed columns indicate the regions transferred from the WKY genome. Vertical lines on the both ends of the columns show intervals including recombination breakpoints. Supplementary Table S3 is available for further information about the candidate genes. Genomic position of each simple sequence repeat marker and SNPs in the *Trpc2*, *Art5* and *Olr111* are defined based on RGSC Genome Assembly v3.4.

SHRSP/Izm and WKY/Izm were provided by the Disease Model Cooperative Research Association (Kyoto, Japan). A congenic strain harboring a WKY-derived small fragment including *Phox2a* and *Ship2* was newly constructed as described previously [5]. Briefly, a congenic strain, harboring the WKY-fragment between D1Smu13 and D1Wox33 [SHRSP.WKY-(D1Smu13-D1Wox33)/Izm (abbreviated as SPwch1.72, see Figure 1)] was backcrossed with SHRSP/Izm, and the resulted F1 rats were intercrossed with one another to obtain F2 rats. Approximately a hundred F2 rats were genotyped to identify a pup with a recombination between D1Smu13 and D1Rat51 (Figure 1). The F2 rat with the recombination was then backcrossed with SHRSP/Izm to obtain male and female heterozygotes, which were mated with each other to establish a new congenic strain with a homozygous WKY fragment only at the D1Smu13 locus [SHRSP.WKY-(D1Smu13)/Izm (abbreviated as SPwch1.71)] (Figure 1). Recombinant breakpoints in each congenic strain were determined by SNP-based genotyping as described below. Male rats of 12 weeks of age were used in all experiments.

### Physiological Evaluation of Stress Responses in Congenic Strains

The responsiveness to restraint and cold stress was examined as described in the previous study [5]; briefly, restraint stress was imposed by placing rats for 3 h in a stainless-steel holder adjusted to the rat’s body size. As for cold stress, a rat was placed in a cage kept at 4°C for 3 h (in the telemetry experiments) or for 6 h (in the collection of urine samples). All experiments for the evaluation of stress response were performed in the afternoon with the light on.

Urinary NE was measured in urine samples by HPLC. The power spectral analysis was done on heart rate variability under restraint stress using the telemetry for ECG (Data Science Inc, St. Paul, MN). The analysis was performed on the ECG using the software implemented in the telemetry system. The ratio between the low frequency (LF; 0.04–1.0 Hz) and the high frequency component (HF; 1.0–3.0 Hz) was used as an indicator of the relative sympathetic activity [5]. The LF/HF ratio was recorded for 30 s in every 10 min throughout the experiment, and the change in LF/HF (ΔLF/HF) was calculated as the difference between the averaged LF/HF during the periods with and without the stresses. Blood pressure (BP) and heart rate (HR) changes under restraint and cold stress were monitored with the telemetry system for BP (Data Science Inc, St. Paul, MN). BP and HR were monitored for 10 s in every 10 min during the experiment. The change in BP (ΔBP) and in HR (ΔHR) was calculated as the difference between the averaged BPs during the periods with and without the stresses.

### Whole Genome Sequencing

Sequencing was performed on AB SOLiD 4 System (Life Technologies, Carlsbad, CA) as previously described [12]. Sequenced libraries were prepared from the whole genome DNA of SHRSP/Izm, SHR/Izm and WKY/Izm according to manufactures instructions using the AB EZ Bead System (Life Technologies, Carlsbad, CA). The size of inserts in fragment and paired-end runs ranged between 210 bp and 300 bp and between 145 bp and 230 bp, respectively. The read length in fragment and paired-end runs was 50 bp and 85 bp, respectively. Five hundred million reads were obtained for the genome of each strain, which covered approximately 20 times the rat genome. The sequence reads were mapped on the Rattus norvegicus genome assembly (rn4) with bowtie (version 0.12.8) [13]. Sequence variations between SHRSP and WKY in the target chromosomal fragments were explored using SAMtools [14].

### mRNA and Protein Expression Analyses

12-weeks old male SHRSP/Izm and WKY/Izm were employed in the experiments. Rats were divided into two groups (N = 5 in each group); one group was kept at a room temperature while the other was exposed to cold stress at 4°C for 6 hours. The rats were then sacrificed under deep anesthesia (by inhalation of diethyl ether) and the brain was dissected quickly. The ventrolateral portion of the brainstem including the rostral ventrolateral medulla (RVLM) was further dissected on ice for RNA and protein extraction.

Total RNA was isolated from the dissected ventrolateral portion of the brainstem using Sepasol-RNA I Super G (Nakalai Tesque, Kyoto, Japan) according to the manufacturer’s instruction. Complementary DNA was synthesized using PrimeScript RT reagent Kit with gDNA Eraser (Takara Bio, Shiga, Japan) with oligo dT primer and random 6mers. Quantitative reverse transcription PCR (RT-PCR) was performed using the Real-time PCR system 7300 (Applied Biosystems, Foster City, CA) in a total volume of 25 µl of reaction mixture using SYBR Premix EX-TaqII (Takara Bio, Shiga, Japan). Quantity of mRNA was standardized on the β-actin mRNA. Primers used in the experiment were listed in Table S1.

Protein samples of the ventrolateral portion of the brainstem were prepared using RIPA buffer (Nakalai Tesque, Kyoto, Japan) containing 50 mmol/L Tris-HCl (pH7.6), 150 mmol/L NaCl, 1.0% Nonidet P-40, 0.5% sodium deoxycholate and 0.1% sodium dodecyl sulfate with a protease inhibitor cocktail, following manufacturer’s instruction. The protein concentration was determined using Protein Assay Bicinchoninate kit (Nakalai Tesque, Kyoto, Japan). Protein samples were separated by SDS-PAGE and transferred to Immobilon-P transfer membrane (Millipore, Billerica, MA). Non-specific binding sites were blocked using PVDF Blocking Reagent (TOYOBO, Osaka, Japan). Western blotting was performed as previously described [15]. ECL Prime Western Blotting Detection Reagent (GE Healthcare, Buckinghamshire, UK) was used for chemiluminescence reaction and the fluorescence image was captured with ImageQuant LAS-4000 (GE Healthcare, Buckinghamshire, UK). Antibodies used in this experiment were as follows: anti-STIM1 (N-terminal) antibody (S6072, 1∶500, Sigma-Aldrich, St. Louis, MO), anti-β-actin (clone AC-15, 1∶2000, Sigma-Aldrich, St. Louis, MO), peroxidase-conjugated goat anti-rabbit IgG polyclonal antibody (1∶2000, Dako, Glostrup, Denmark), and peroxidase-conjugated sheep anti-mouse IgG polyclonal antibody (GE Healthcare, Buckinghamshire, UK). All antibodies used in this experiment were diluted in Can Get Signal solution (TOYOBO, Osaka, Japan).

### Single Nucleotide Polymorphism (SNP)-based Genotyping

DNA samples of W/KYO, WKYO/Kyo, WKY/Crj, SDJ/Hok, LEJ/Hok, SS/JrNgs and SR/JrNgs were provided through the National Bio-Resource Project for Rats [16]. Genomic DNA of other rat strains listed below were extracted from frozen liver tissues or blood using Gentra puregene kit (Qiagen, Hilden, Germany) or DnaQuick II (DS Pharma Biomedical, Osaka, Japan) according to the manufacturer’s instruction. Examined strains were as follows; SPwch1.71 and SPwch1.72 (constructed as described above), LEW/SsNslc and F344/SsNslc (purchased from SLC, Hamamatsu, Japan), SHRSP/Izm, WKY/Izm, SHRB2, SHRCH, SHRCL, SHRSPA1-sb and SHRSPA4 (provided by the Disease Model Cooperative Research Association, Kyoto, Japan), SHR/Kyushu (provided by Dr. Setsuro Ibayashi), SHRSP/Ngsk (provided by Dr. Masami Niwa) and SHRSP/Ezo (provided by Dr. Hiroko Togashi). SNP typing was performed by direct sequencing of PCR products amplified with primers listed in Table S2 using BigDye terminator v.1.1 (Applied Biosystems, Foster City, CA) and the 3130 Genetic Analyzer (Applied Biosystems, Foster City, CA).

### Statistics

Means and standard deviations are shown in the figures, tables and the text. Inter-strain differences were tested either by Student’s t-test or by ANOVA with Dunnett’s post-hoc test. *P*<0.05 was considered to be statistically significant.

## Results

### Physiological Evaluation of the Congenic Strains

Effects of cold and restraint stress on BP, HR, ΔLF/HF and urinary NE excretion were summarized in Table 1.

**Table 1:** Evaluation of stress response in the congenic strains.

	Strain			
Parameters	SHRSP	SPwch1.71	SPwch1.72	WKY
BP (mmHg)	213±31 (4)	209±19 (4)	212±24 (4)	111±10*(6)
*Δ*BP (mmHg), Restraint	22.4±11.8 (4)	33.8±8.1 (4)	2.0±3.3*(4)	4.1±5.7*(6)
Cold	34.4±4.3 (4)	48.1±17.7 (4)	15.7±6.1*(4)	17.3±8.1*(6)
HR (bpm)	331±71 (3)	272±21 (4)	348±40 (4)	343±18 (6)
*Δ*HR (bpm), Restraint	67.2±18.4 (3)	100.1±20.9 (4)	102.1±10.9*(4)	33.3±20.3*(6)
Cold	179.0±32.6 (3)	189.2±27.0 (4)	96.9±60.0 (4)	127.1±20.0*(6)
*Δ*LF/HF, Restraint	3.38±0.51 (5)	3.35±1.00 (5)	1.92±1.11*(5)	0.34±0.30*(5)
*Δ*NE (µmol), Cold	0.572±0.096 (10)	0.596±0.061 (10)	0.341±0.046*(10)	0.173±0.069*(16)

Baseline blood pressure (BP), heart rate (HR), increase in BP (*Δ*BP), HR (*Δ*HR), LF/HF (*Δ*LF/HF, a parameter for the relative sympathetic activity), and changes in urinary norepinephrine excretion (*Δ*NE) under cold and restraint stress in SHRSP were compared with those in SPwch1.71,SPwch1.72 and WKY [5]. The numbers of rats used for each analysis are shown in parentheses. *P<0.05 vs. SHRSP by Dunnett’s post-hoc test.

BP of SPwch1.71 did not differ significantly from that of SHRSP when measured by the telemetry method (Table 1). When BP change under the cold and restraint stress was monitored by the telemetry, the response in SPwch1.71 was not significantly different from that in SHRSP, while SPwch1.72 showed blunted response that was comparable with that in WKY (Table 1). In accordance with this observation, increase in urinary NE excretion under the cold stress did not differ significantly between SPwch1.71 and SHRSP, while the response in SPwch1.72 was significantly less than that in SHRSP (Table 1). Further, a power spectral analysis on HR variation indicated that ΔLF/HF, a parameter of the relative sympathetic activity [5], under the restraint stress was comparable between SHRSP and SPwch1.71, whereas a significant reduction was observed in SPwch1.72 (Table 1).

Collectively, the results of these physiological experiments indicated that the region covered by SPwch1.71 did not contribute to the difference in the sympathetic stress response between SHRSP and WKY, and could be excluded from the candidate region.

Considering that results of ΔHR under the stress were inconsistent with the other three parameters, we suspected that, in contrast to the power spectral analysis on HR variation, ΔHR itself did not seem a good parameter to measure the inter-strain difference (Table 1); it might be too sensitive to various environmental noises.

Although influence of physical activity on HR and BP could be excluded in the restraint stress experiments (rats could not move at all), it might influence HR and BP in the cold stress experiments. In the present study, we could not monitor the physical activity of rats because of a technical difficulty, however, we expected the influence was small because a small size of cages used in the experiments did not give rats enough space to move around. This was further supported by the fact that the results of the restraint and the cold stress experiments were consistent with each other.

### Refinement of the Boundary of the Candidate Region

According to the physiological studies above, a gene (or genes) responsible for the exaggerated sympathetic response in SHRSP seemed to be in the region covered not by SPwch1.71 but by 1.72 (see Figure 1). To determine the boundaries of the target region, the recombinant breakpoints of the congenic regions in SPwch1.71 and in SPwch1.72 were refined. By genotyping of single-nucleotide polymorphisms (SNPs) located between D1Smu13 and D1Got154 (SNPs used in the genotyping are listed in Table S2), we identified that the recombinant breakpoint of the congenic fragment in SPwch1.72 at the telomeric side was located between D1Wox33 and a SNP in the coding region of the *Olr111* gene, and that of the fragment in SPwch1.71 was between SNPs in the *Trpc2* and in the *Art5* gene, respectively (Figure 1 and Table S2). As a result, we narrowed down the candidate region between the SNP in *Art5* and D1Wox33 as the minimal estimation (1.2-Mbp) or between the SNPs in *Trpc2* and in *Olr111* as the maximal estimation (1.8-Mbp) (Figure 1).

### Evaluation of Candidate Genes

**Figure 2:**
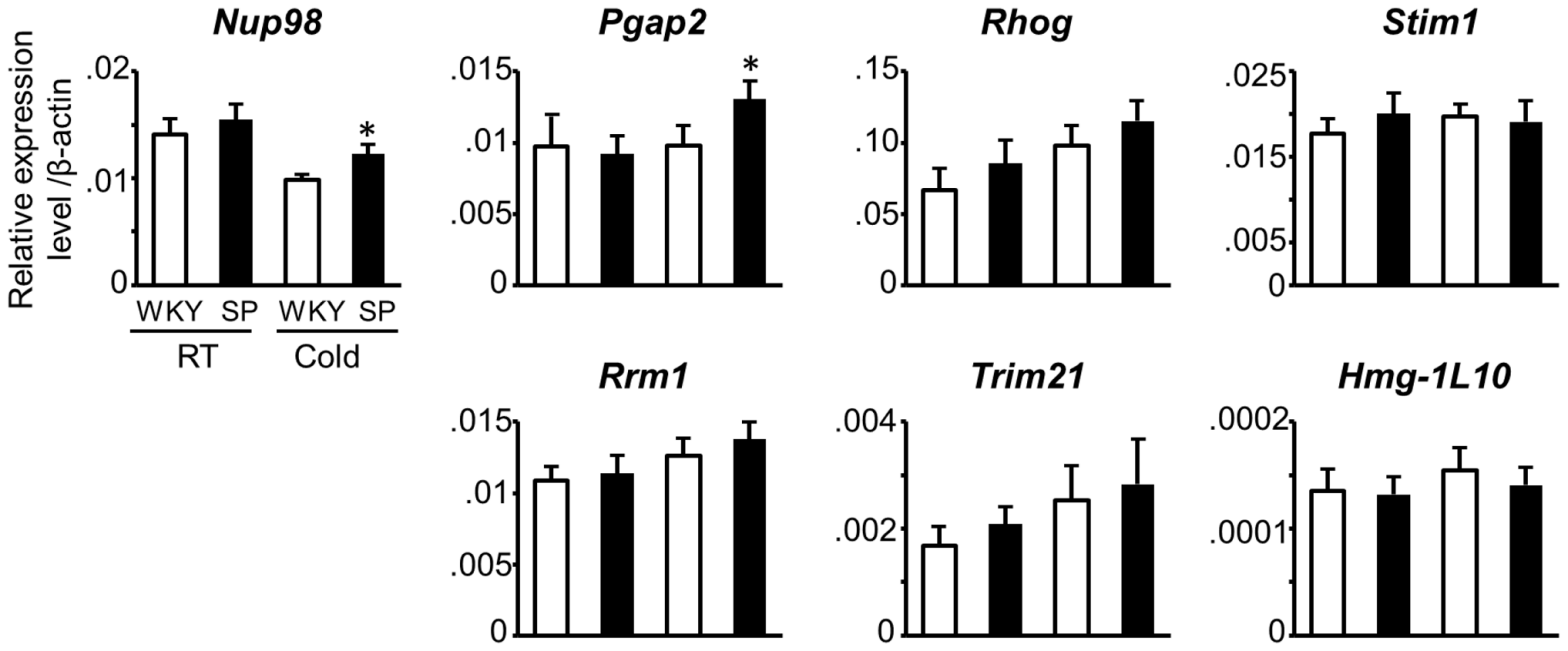
Expression analysis of candidate genes in the brainstem of WKY and SHRSP. Five rats of the each strain were used. Ventrolateral part of the brainstem including RVLM was dissected and RNA was extracted. Relative levels of gene expression were evaluated by quantitative RT-PCR analysis. The expression levels of each gene were normalized with β-actin mRNA. Each column shows the expression level of WKY and SHRSP under the room temperature (RT) or under the cold stress (Cold) as indicated in the panel for *Nup98*. SP: SHRSP, *P<0.05 vs. WKY under the cold stress by Student’s t-test.

In 1.8-Mbp of the maximally estimated candidate region, we identified 69 annotated genes, 2 predicted genes and 18 pseudogenes. Among the annotated genes, 59 were genes for olfactory receptors. After excluding the pseudogenes and the genes for olfactory receptors, 12 genes (10 were annotated) were located in this region (Figure 1 and Table S3). We compared the expression level of the 12 genes in the brainstem between WKY and SHRSP. The expression of *Trpc2*, *Art5*, *Art1*, *Chrna10* and *LOC685521* were minimal or absent, and thus were excluded from the candidate genes (data not shown). Among the rest of 7 candidate genes, none showed a significant difference in the basal expression between the two strains. Under the cold stress, on the other hand, *Nup98* and *Pgap2* showed a modest, but statistically significant, difference in the gene expression between SHRSP and WKY (Figure 2).

The whole-genome sequence analysis of WKY/Izm and SHRSP/Izm identified nonsynonymous single-nucleotide substitutions in three genes, *Art1*, *Stim1* and *Trim21*, of the 12 candidate genes (Table 2). *Art1* was, however, excluded because the expression was not detectable in the brainstem (see above). In *Trim21*, we identified two nonsynonymous nucleotide substitutions (Table 2). One of them, the A to G substitution at 160,175,885 bp (p.Ile474Met) was found in WKY/Izm while SHRSP/Izm had the wild-type allele. As the two strains did not share the same allele, this missense substitution might cause phenotypic differences between SHRSP and WKY.

**Table 2:** Nonsynonymous substitutions identified in candidate genes located in a 1.8-Mbp region on chromosome 1.

Genomic position		Nucleotide and amino acid			
(bp)*	Gene	Reference	WKY/Izm	SHR/Izm	SHRSP/Izm
15,95,90,430	*Art1*	C (Arg)	C (Arg)	T (STOP)	T (STOP)
15,99,15,391	*Stim1*	G (Leu)	C (Phe)	G (Leu)	G (Leu)
15,99,24,291	*Stim1*	C (Arg)	C (Arg)	C (Arg)	T (STOP)
16,01,75,885	*Trim21*	A (Ile)	G (Met)	A (Ile)	A (Ile)
16,01,76,069	*Trim21*	A (Gln)	A (Gln)	T (Leu)	A (Gln)

*The genomic position of SNP is defined based on the RGSC Genome Assembly v3.4.

**Figure 3:**
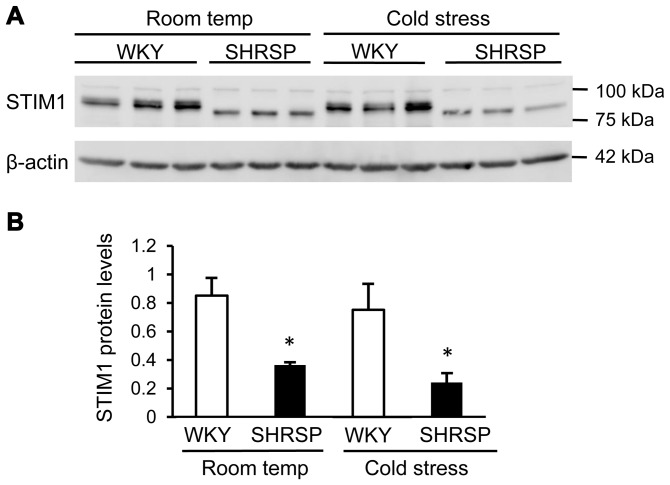
Western blot analysis of STIM1. A) Western blotting was performed as described in the Materials and Methods. The size of STIM1 in SHRSP was obviously smaller than that in WKY. A representative data is shown. B) Semi-quantitative evaluation of the STIM1 protein level was performed using ImageJ software. The relative amount of STIM1 was standardized with the level of β-actin. Room temp: room temperature, *P<0.05 vs. WKY by Student’s t-test.

Eventually, four genes, *Stim1*, *Nup98*, *Pgap2* and *Trim21*, remained putative candidate genes. Among them, *Stim1* was the most promising candidate because of the functional role (see Discussion) and of a nonsense mutation found in the 3′-end of the coding region in SHRSP (Table 2). As expected from the sequence analysis, a western blot analysis revealed that a truncated form of STIM1 was expressed in the brainstem of SHRSP (Figure 3A). In addition, the level of the STIM1 protein was significantly lower in SHRSP than in WKY regardless of whether exposed to cold stress or not (Figure 3A and 3B).

Genotyping of the two variations in *Stim*1 was performed in seventeen rat strains including 3 substrains of WKY, 4 of SHR and 4 of SHRSP, which showed that the stop codon responsible for the truncation was identified only in 4 substrains of SHRSP (except SHRSPA1-sb) and 1 substrain of SHR, i.e., SHR/Kyushu (Table 3). Neither WKYs nor other major laboratory rat strains shared this nonsense mutation (Table 3).

**Table 3:** Allelic difference of the two sequence variations identified in *Stim1* in various rat strains.

	Position and nucleotide	
Strains	159,915,391 bp	159,924,291 bp
WKY/Izm	C	C
W/Kyo	G	C
WKYO/Kyo	G	C
WKY/Crj	C	C
SHR/Izm	G	C
SHR/B2	G	C
SHR/CL	G	C
SHR/Crj	G	C
SHR/Kyushu	G	T
SHRSP/Izm	G	T
SHRSPA1-sb	G	C
SHRSPA4	G	T
SHRSP/Ngsk	G	T
SHRSP/Ezo	G	T
SDJ/Hok	G	C
LEJ/Hok	G	C
F344/NSlc	G	C
LEW/SsNSlc	G	C
SS/JrNgS	G	C
SR/JrNgS	G	C

Genomic position of each SNP is defined based on RGSC Genome Assembly v3.4.

We found another nonsynonymous substitution (p.Leu488Phe) in STIM1 that was specific for WKY/Izm and WKY/NCrj, which may have functional significance as well (Table 2 and Table 3).

## Discussion

The previous study indicated that the congenic region covered by SPwch1.72 harbored a gene (or genes) responsible for the exaggerated stress response in SHRSP [5]. In the present study, we further narrowed down the region to a 1.2-Mbp fragment, and identified a new candidate, *Stim1*, among the genes located in this region.

In the region covered by SPwch1.72, we initially found a strong functional candidate gene, *Phox2a*, a transcription factor regulating the expression of *Th* as well as the development of SNS *in utero* [6], [7]. We therefore attempted to make another congenic strain (SPwch1.71) covering a small fragment including *Phox2a* (Figure 1). The physiological evaluation of this congenic strain indicated that the region harboring *Phox2a* could be excluded from the region responsible for the difference in the stress response between SHRSP and WKY (Table 1 and Figure 1). The refinement of the congenic boundaries using newly identified SNPs in this region excluded *Ship2* from the candidate genes as well (Figure 1).

Although the two candidate genes were excluded, we identified another candidate gene, *Stim1*, in the newly defined target region, which harbored a nonsense mutation (p.Arg640X) in SHRSP (Table 2). STIM1 is a Ca^2+^-storage sensor protein localized on the endoplasmic reticulum (ER) and plasma membrane, and is an essential component in the store-operated calcium entry (SOCE) process through regulating a calcium channel, ORAI1, on the cell membrane [17]–[21]. The SOCE was found in various types of cells, including neurons, and shown to play important roles in a variety of cellular functions [22], [23]. The nonsense mutation found in SHRSP resulted in a truncation of 46 amino acids at the C-terminal end of the rat STIM1 that was indeed shown by the western blot analysis with anti-STIM1 (N-terminal) polyclonal antibody (Figure 3A). The specificity of the analysis was confirmed with another anti-STIM1 monoclonal antibody (Abnova, clone 5A2, data not shown).

Of note, it is suggested that the C-terminus lysine residues (K684, K685), which were lost in the truncated form, were essential in interaction of STIM1 with the transient receptor potential cation channel 1 (TRPC1), another type of cation channels on the cell membrane [24]. As STIM1 was postulated to control the gating of TRPC1, it is attractive to hypothesize that the truncation in STIM1 is causally related to the exaggerated response of SNS in SHRSP through abnormal regulation of TRPC1. In addition, Bauer et al. showed that calmodulin bound to the polybasic C-terminal of STIM1 in a calcium-dependent manner [25]. This implied that the lack of the C-terminal residues of STIM1 affected the calmodulin-dependent regulation of STIM1 as well.

In addition to the truncation, we also found the protein level of STIM1 in the brainstem was lower in SHRSP when compared with WKY although the mRNA expression level was not significantly different (see Figure 2 and Figure 3). Keil et al. raised a possibility that the ubiquitin-proteasome system regulated the STIM1 protein level when the SOCE was activated [26]. The truncated STIM1 might be degraded more rapidly in this system, which might resulted in the decreased level of STIM1 in SHRSP. The difference in the protein level *per se* may have a functional significance even if the truncation is pathologically innocent.

In this context, it is of note that Giachini et al. showed that augmented activity of STIM1 and ORAI1 in the aorta of SHRSP resulted in greater SOCE-dependent vasoconstriction [27]. They found that the protein expression of both STIM1 and ORAI1 was increased in the aorta of SHRSP when compared with WKY. Further, they revealed that the SOCE inhibitors (2-aminoethoxydiphenyl borate and gadolinium) as well as neutralizing antibodies against STIM1 and ORAI1 abrogated the SOCE-dependent vasoconstriction observed in SHRSP. Their findings indicated that augmented activity of STIM1 and ORAI1 might be causally related to hypertensive phenotype in SHRSP. As far as our results of western blotting are concerned, however, the present results are inconsistent with those in the report by Giachini et al. in terms of the molecular size and the expression level of STIM1 in SHRSP (see Figure 3). Although we do not have a good interpretation on this discrepancy, the different source of SHRSP used in the experiments might affect the results. In fact, we found that the allele of *Stim1* in SHRSPA1-sb (a substrain of SHRSP) differed from that in SHRSP/Izm (Table 3). In addition, different tissue used in the experiments (i.e., the brainstem vs. the aorta) might explain the discrepancy in the expression level of STIM1 between the two reports.

In anyway, difference in the STIM1 protein expression, which was a rather unexpected result, must be confirmed between SPwch1.71 and 1.72 to obtain more robust evidence on influence of the *Stim1* genotype on the protein expression.

The fact that the substrains of SHRSP shared the nonsense mutation in *Stim1* implicated an important role of this gene in generation of SHRSP-specific phenotype. However, we had two exceptions, i.e., SHR/Kyushu and SHRSPA1-sb; SHR/Kyushu, a substrain of SHR, shared the same allele with SHRSP/Izm, while SHRSPA1-sb did not (Table 3). According to the original study describing the establishment of SHRSP, SHRSPA1-sb was developed in parallel with SHRSPA3 (which is now called SHRSP/Izm), and showed lower BP than that in SHRSPA3 [28]. In addition, SHR/Kyushu was a descendant of the “original” SHR that was not yet separated from SHRSP, and therefore was expected to share some phenotypic and genotypic features with SHRSP. In fact, Cai et al. showed that SHR/Kyushu was more vulnerable to the middle cerebral artery occlusion to give larger infarction areas when compared with SHR/Izm [29].

In this context, evaluation of the sympathetic stress response between SHR/Izm and SHR/Kyushu may provide further evidence on the influence of the truncated STIM1 on the sympathetic stress response. We therefore compared urinary NE excretion under the cold stress between the two SHR substrains (Figure S1). The excretion in SHR/Kyushu (with the SHRSP allele of *Stim1*) tended to be greater when compared with SHR/Izm (with the wild-type allele of *Stim1*), though the difference did not reach a significant level (*P* = 0.088). As SHR/Kyushu shared the same genetic background with SHRSP in substantial areas of Chr1, other genes in the background might modify the result [12]. The influence of the genetic background other than *Stim1* on the urinary NE excretion was further supported by the fact that SPwch1.72, which shared the same allele of *Stim1* with WKY, showed significantly greater NE excretion than that in WKY (*P*<0.001, see Table 1).

Another missense variation specific for WKY/Izm and WKY/NCrj may have functional importance (Table 3); Mullins et al. reported that a short domain of STIM1 (residues 470–491) was required for Ca^2+^-dependent inactivation of ORAI1 [30]. This finding implies that the p.Leu488Phe mutation identified in the two substrains of WKY may influence the SOCE activity via interaction with ORAI1.

*Nup98* and *Pgap2* were potential candidate genes because the significant differences in the gene expression under the cold stress were observed between SHRSP and WKY (Figure 2). To our knowledge, promoter sequences or transcription factors regulating the expression of *Nup98* and *Pgap2* remains to be determined. Based on the sequence analysis of SHRSP and WKY, we found three sequence variations in the 5′-untraslational region of the two genes; a G to T substitution at 1,836 bp upstream from the first exon of *Nup98*, and a C to T and a T to C substitution at 1,729 bp and 4,390 bp upstream from the first exon of *Pgap2*, respectively. We thus searched for possible transcription factors bound to the genomic sequences including the three substitutions above using the TRANSFAC MATCH v.1.0. The results indicated that no such transcription factors were so far identified, and the functional significance of the three substitutions was unclear.

Further, as far as the gene functions currently annotated are considered, these two genes do not seem strong candidates for the genes regulating sympathetic nervous activity; *Pgap2* encodes a Goldi/ER-resident membrane protein and is involved in fatty acid glycosylphosphatidylinositol (GPI)-anchor remodeling, which is required for stable association of GPI-anchored proteins and the cell-surface membrane rafts [31], [32]. NUP98 is a member of nucleoporins and the formation of fusion genes with various partner genes was reported to be associated with hematopoietic malignancy [33]. In spite of the discussion above, to obtain further clues for the candidacy, the expression of these genes in SPwch1.71 and 1.72 needs to be examined in a future study. In addition, although differences in the expression of *Nup98* and *Pgap2* between WKY and SHRSP were statistically significant, the difference was rather modest (1.2 and 1.3 fold, respectively, see Figure 2) and requires confirmation at the protein level.

*Trim21* remained to be a putative candidate gene because of the A to G nonsynonymous substitution (p.Ile474Met) was identified between WKY and SHRSP (Table 2). TRIM21, also known as Ro52, has the E3 ligase activity involved in the ubiquitination process and it was reported that autoantibodies against this protein were detected in patients with several autoimmune diseases, e.g., primary Sjögren’s syndrome [34]. As in the case of NUP98 and PGAP2, the function of TRIM21 currently annotated does not strongly infer roles of this gene in the sympathetic stress response.

Based on the discussion above, at the moment, we concluded that *Stim1* was the most promising among the four putative candidates. In spite of that, careful further evaluation of the other three genes is essential before they are excluded from candidate genes for sympathetic stress response.

In conclusion, we found that *Stim1* is the best candidate in terms of the gene function as well as of the potential significance of the sequence variations identified in it. Accumulating evidence suggests that STIM1 is involved in various pathophysiological processes such as inflammatory immune response, cardiac hypertrophy and hypertension [27], [35], [36]. In spite of that, roles of STIM1 and TRPCs in normal and in pathological conditions are not fully elucidated. To obtain conclusive evidence for the causative role of the truncated STIM1, it is essential to clarify effects of the truncation (or the low expression) on the cellular calcium dynamics. Further studies on the role of STIM1 in the regulation of SNS are warranted.

## Supporting Information

Figure S1 **Evaluation of sympathetic response to cold stress in SHR/Izm and SHR/Kyushu.** A rat was place in a metabolic cage kept at 4°C for 6 hours. An urine sample was collected for HPLC analysis of urinary norepinephrine excretion (NE) and changes in urinary NE (ΔNE) under cold stress was evaluated. Nine rats of the each strain were used for the experiment. *P* value was calculated by Student’s t-test and shown at the top of columns.
(PPTX)

Table S1 **Primer sequences for quantitative RT-PCR analysis.**
(PPTX)

Table S2 **Primer sequences for SNP-based genotyping.** UTR; untranslated region, CDR; coding region. *The genomic position of each SNP is defined based on the RGSC Genome Assembly v3.4.
(PPTX)

Table S3 **Putative genes in the 1.8-Mbp congenic fragment.**
(PPTX)
